# Mechanistic computational modeling of sFLT1 secretion dynamics

**DOI:** 10.1101/2025.02.12.637983

**Published:** 2025-02-18

**Authors:** Amy Gill, Karina Kinghorn, Victoria L. Bautch, Feilim Mac Gabhann

**Affiliations:** 1 Institute for Computational Medicine, Johns Hopkins University, Baltimore, MD, USA; 2 Department of Biomedical Engineering, Johns Hopkins University, Baltimore, MD, USA; 3 Department of Biology, University of North Carolina, Chapel Hill, NC, USA; 4 Curriculum in Cell Biology and Physiology, University of North Carolina, Chapel Hill, NC, USA; 5 McAllister Heart Institute, University of North Carolina, Chapel Hill, NC, USA

## Abstract

Constitutively secreted by endothelial cells, soluble FLT1 (sFLT1 or sVEGFR1) binds and sequesters extracellular vascular endothelial growth factors (VEGF), thereby reducing VEGF binding to VEGF receptor tyrosine kinases and their downstream signaling. In doing so, sFLT1 plays an important role in vascular development and in the patterning of new blood vessels in angiogenesis. Here, we develop multiple mechanistic models of sFLT1 secretion and identify a minimal mechanistic model that recapitulates key qualitative and quantitative features of temporal experimental datasets of sFLT1 secretion from multiple studies. We show that the experimental data on sFLT1 secretion is best represented by a delay differential equation (DDE) system including a maturation term, reflecting the time required between synthesis and secretion. Using optimization to identify appropriate values for the key mechanistic parameters in the model, we show that two model parameters (extracellular degradation rate constant and maturation time) are very strongly constrained by the experimental data, and that the remaining parameters are related by two strongly constrained constants. Thus, only one degree of freedom remains, and measurements of the intracellular levels of sFLT1 would fix the remaining parameters. Comparison between simulation predictions and additional experimental data of the outcomes of chemical inhibitors and genetic perturbations suggest that intermediate values of the secretion rate constant best match the simulation with experiments, which would completely constrain the model. However, some of the inhibitors tested produce results that cannot be reproduced by the model simulations, suggesting that additional mechanisms not included here are required to explain those inhibitors. Overall, the model reproduces most available experimental data and suggests targets for further quantitative investigation of the sFLT1 system.

## Author Summary

Proteins that are typically found outside cells are initially made inside cells, and later secreted into extracellular space. Many of these secreted proteins have important functions outside the cell that are well-studied; however, usually much less is known about the pre-secretion life of these molecules. Many computational models only represent the extracellular versions of secreted proteins, reducing all production and secretion steps into a single modeled process. Here, we develop a mechanistic model of the production and secretion of a specific secreted protein, sFLT1, which inhibits blood vessel growth by acting as an extracellular sponge for another set of secreted proteins, the vascular endothelial growth factors. We compare several models to existing experimentally-measured sFLT1 data, and we show that the data are most simply explained by including a delay between intracellular sFLT1 production and sFLT1 transport or degradation. This is consistent with the biology of the cell's secretory pathway, where immature proteins are gradually processed into mature forms over minutes to hours. Our approach could be incorporated into improved models for any pathway involving secreted proteins, including sFLT1-regulated models of blood vessel biology.

## Introduction

Secreted proteins, which represent 15% of the human genome and 9% of the human proteome, are essential local and systemic signaling factors in multicellular systems.^1^ Most proteins destined for the extracellular space, or for intracellular locations other than the cytoplasm and nucleus, mature via the secretory pathway, in which new proteins are cotranslationally translocated into the endoplasmic reticulum (ER), folded, transported to the Golgi apparatus, post-translationally modified, sorted, and trafficked by vesicles to target compartments.^2^ Many proteins synthesized through the secretory pathway are released by exocytosis, while others reside and function mainly in intracellular compartments (such as the Golgi or endosomes).^3–5^ Many proteins known to function extracellularly or at the plasma membrane also have biologically meaningful interactions in intracellular compartments, including receptor tyrosine kinases.^6–10^ Although all secreted proteins begin as intracellular proteins, intracellular roles and dynamics of extracellularly secreted proteins are generally understudied.

Here, we explore the secretion of soluble FLT1 (sFLT1, sVEGFR1), a key negative regulator of the vascular endothelial growth factors (VEGF), a family of cytokines that promote angiogenesis. sFLT1 is an alternatively spliced truncated isoform of VEGF Receptor 1 (VEGFR1 or FLT1) that retains the extracellular domains for binding ligands (including VEGFA, VEGFB, and placental growth factor (PlGF)) and heparan sulfate proteoglycans (HSPGs), but lacks transmembrane or intracellular tyrosine kinase domains, yielding a secreted, non-membrane anchored, and non-signaling receptor.^11,12^ Constitutively secreted by endothelial cells,^13^ sFLT1 binds and sequesters most extracellular VEGF isoforms, thereby reducing binding of these VEGF isoforms to VEGF receptor tyrosine kinases and the downstream signaling.^11,14–16^ As a VEGF antagonist and antiangiogenic factor, sFLT1 prevents vascular overgrowth and is essential for corneal avascularity.^17^ This inhibitory role is also important during angiogenesis for proper patterning of new blood vessel networks: sFLT1 from endothelial cells interacts extracellularly with stromal VEGF to reinforce VEGF gradients extending away from existing vasculature, guiding nascent vascular sprouts to reduce vascular overgrowth and disorganization.^18–20^

Dysregulated sFLT1 production and secretion is a pathogenic factor in several disorders including preeclampsia, a syndrome of endothelial dysfunction during pregnancy characterized by hypertension, kidney damage, and significant maternal and fetal morbidity and mortality. In preeclampsia, excessive sFLT1 production and secretion from the placenta leads to elevated serum sFLT1, reducing free serum levels of VEGF and PlGF, inhibiting VEGF-mediated vasodilation and angiogenesis, causing hypertension and damaging vasculature.^21^ While we do not focus on pathology here, a quantitative understanding of sFLT1 production and secretion from endothelial cells could be extended in the future to simulate pathologies.

While the extracellular role of sFLT1 is well characterized, less is known about its intracellular biology. Recent work suggests unexpectedly high intracellular sFLT1 levels in endothelial cells and long intracellular residence time,^13,22^ motivating a quantitative characterization of sFLT1 trafficking and elucidation of the intracellular dynamics of sFLT1 and its possible intracellular roles. Past studies also suggest the full-length membrane-integral FLT1 (mFLT1) protein resides largely intracellularly,^23^ much of it in the Golgi, and it traffics to the plasma membrane in a calcium-dependent manner.^8^ sFLT1 may be retained in secretory organelles by a similar mechanism to the larger mFLT1 isoform.

Mechanistic computational modeling represents biochemical molecular networks as systems of equations based on prior knowledge of regulatory interactions and parameterized with experimental data. These models allow virtual exploration of molecular networks while tracking components that may be difficult to measure experimentally, and are powerful tools for testing mechanistic hypotheses and screening new treatment approaches.^24^ While many mechanistic models describe systems as sets of ordinary differential equations (ODEs), time delays between processes are common in biology, and recent work shows that delay differential equations (DDEs) can better represent the behavior of several common network motifs in biochemical reaction networks.^25^ This study builds on our recent model of sFLT1 production and secretion in endothelial cells,^13^ which we believe is the first mechanistic model to include intracellular sFLT1. Several previous models of the VEGF system have included the impact of sFLT1 secretion on extracellular sFLT1 and VEGF levels, including pharmacological-like distribution across tissues^26–29^ and local gradients,^19^ but did not explore the intracellular dynamics of sFLT1 secretion. In our recent work characterizing intracellular sFLT1 trafficking from protein synthesis to secretion,^13^ we briefly introduced a model linking intracellular and extracellular sFLT1 pools through protein synthesis, secretion, and intracellular and extracellular degradation. We used this model to estimate a basal secretion rate of ~30,000 molecules per cell per hour in human umbilical vein endothelial cells (HUVEC), comparable to other secreted proteins, and to infer that Golgi trafficking is absolutely required for sFLT1 secretion.^13^

Here, we extend our previous work to develop multiple mechanistic models of sFLT1 secretion and to identify a minimal mechanistic model that recapitulates key qualitative and quantitative features of experimental sFLT1 time course datasets. We compare several ODE-based and DDE-based models to capture the dynamics of sFLT1 secretion, using experimental data from multiple studies to parameterize the models and evaluate their relative performance. We then compare simulation predictions of perturbation by secretion pathway inhibitors to the outcomes of equivalent experiments.

## Methods

### Models of sFLT1 production and secretion.

We constructed several mechanistic models to explore sFLT1 secretion dynamics, each of which consists of two coupled nonlinear differential equations describing the levels of sFLT1 in two locations: extracellular sFLT1 $\left(X\right)$ that has been secreted out of the cell into conditioned media, and intracellular sFLT1 $\left(I\right)$, a sum of immature and mature protein inside the cell, treating all intracellular locations (ER, Golgi, vesicles, etc.) as one well-mixed compartment. Molecular levels of I and X were tracked in units of number per cell (#/cell). We briefly introduced one example of these models in recent work;^13^ here we provide additional detail on model construction and analysis, compare it to additional model formulations, and re-parameterize based on additional experimental data.

### ODE-based model.

**Figure 1.**
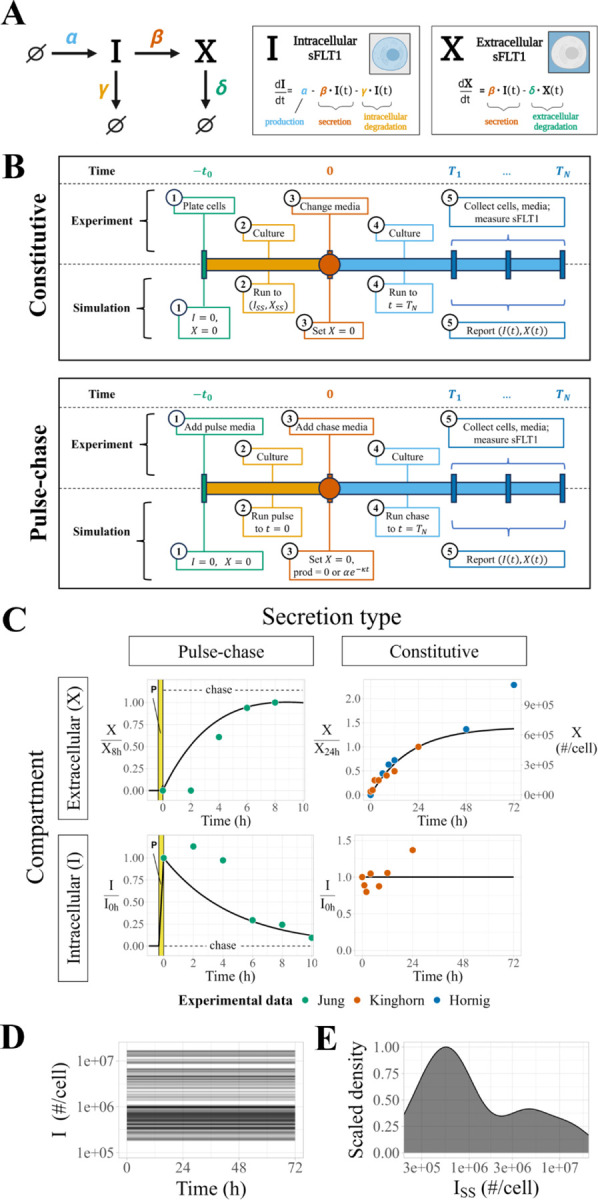
Modeling sFLT1 secretion with ordinary differential equations (ODEs). **(A)** Model of sFLT1 secretion dynamics describing the impact of production (rate α), secretion (rate constant β), intracellular degradation (γ), and extracellular degradation (δ) on the concentrations of intracellular $\left(I\right)$ and extracellular $\left(X\right)$ sFLT1 as a system of ordinary differential equations (ODEs). Created with Biorender.com. **(B)** Comparison of simulation (top row) and experimental (bottom row) approaches for studying sFLT1 secretion in the constitutive secretion scenario (left column) and pulse-chase secretion scenario (right column). **(C)** Simulated time courses $\left(n=100\right)$ of extracellular (X, top) and intracellular (I, bottom) sFLT1 secretion for pulse-chase (left) and constitutive (right) cases using the ODE model. X was normalized to its value at 8h (upper left) or 24h (upper right), while I was normalized to its value at 0h. The highlighted region (P) marks the 20-minute pulse. **(D)** Number of intracellular sFLT1 $\left(I\right)$ molecules per cell over time and **(E)** distribution of steady state intracellular sFLT1 $\left(I_{SS}\right)$ during simulations of constitutive secretion using the ODE model.

We created an initial model using ordinary differential equations (ODEs) (Figure 1A) to describe how the mechanistic processes impact the level of intracellular and extracellular sFLT1. The equations for intracellular and extracellular sFLT1 include rates for four mechanistic processes:

- *production*, the synthesis of new intracellular protein $\left(\emptyset \to I\right)$ characterized as a zeroth-order process with a rate constant α (when on) or 0 (when off), representing translation of intracellular protein and assuming no feedback dependence of sFLT1 production on current sFLT1 levels;
- *secretion*, the transport from the intracellular to extracellular space $\left(I\to X\right)$ characterized as a first order process with rate constant β, representing exocytosis of mature sFLT1 protein;
- *intracellular degradation*, the depletion of intracellular sFLT1 $\left(I\to \emptyset \right)$ characterized as a first order process with rate constant γ, representing lysosomal degradation or other proteolytic degradation mediated by intracellular factors;
- *extracellular degradation*, the depletion of extracellular sFLT1 $\left(X\to \emptyset \right)$ characterized as a first order process with rate constant δ, representing proteolytic degradation mediated by soluble, membrane-bound, or matrix-bound extracellular factors.

Thus, the following equations define the time evolution of the system:

(Eq. 1) $$\frac{dI\left(t\right)}{dt}=\text{α}-\text{β}\cdot I\left(t\right)-\text{γ}\cdot I\left(t\right)$$

(Eq. 2) $$\frac{dX\left(t\right)}{dt}=\text{β}\cdot I\left(t\right)-\text{δ}\cdot X\left(t\right)$$

### DDE-based model.

**Figure 2.**
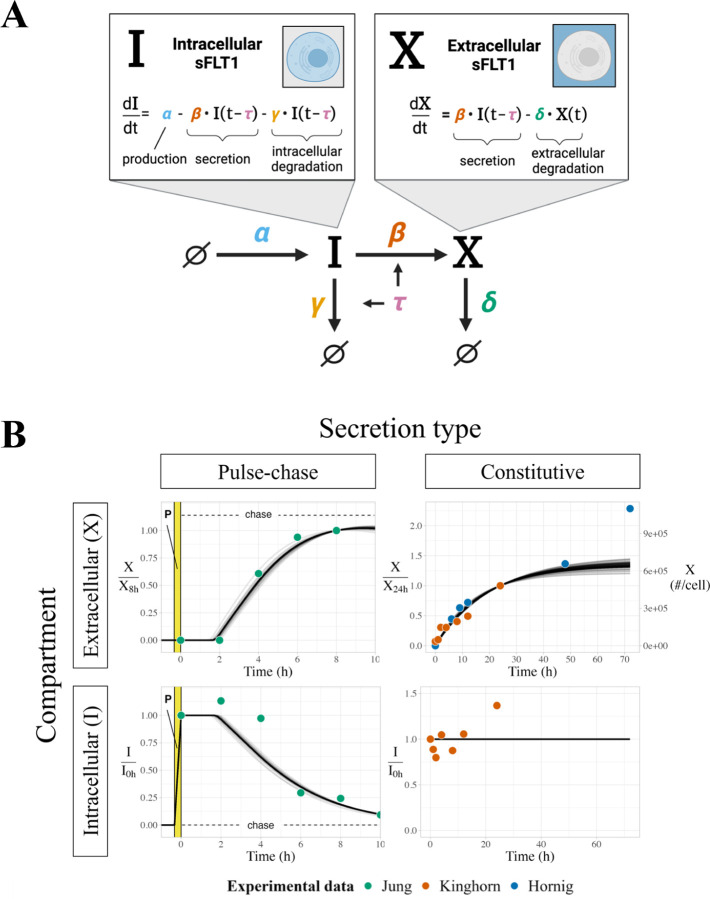
Modeling sFLT1 secretion with delay differential equations (DDEs). **(A)** Model of sFLT1 secretion dynamics as a system of delay differential equations (DDEs), extending the ODE model (Figure 1A) with a fixed maturation delay (τ) affecting secretion and intracellular degradation processes. Created with Biorender.com. **(B)** Simulated time courses $\left(n=594\right)$ of extracellular (X, top) and intracellular (I, bottom) sFLT1 secretion for pulse-chase (left) and constitutive (right) cases using the base DDE model. X was normalized to its value at 8h (upper left) or 24h (upper right), while I was normalized to its value at 0h. The highlighted region (P) marks the 20-minute pulse.

We converted our initial ODE model to a system of delay differential equations (DDEs) (Figure 2A) by introducing a fixed time delay τ to the secretion and intracellular degradation processes, representing a required protein maturation period before I can be transported or degraded:

(Eq. 3) $$\frac{dI\left(t\right)}{dt}=\text{α}-\text{β}\cdot I\left(t-\text{τ}\right)-\text{γ}\cdot I\left(t-\text{τ}\right)$$

(Eq. 4) $$\frac{dX\left(t\right)}{dt}=\text{β}\cdot I\left(t-\text{τ}\right)-\text{δ}\cdot X\left(t\right)$$

### Candidate mechanistic models.

We formulated eight distinct candidate models containing different combinations of 3 processes:

- *maturation delay*, the fixed time delay τ described above affecting secretion and intracellular degradation
- *internalization,* a first order transport reaction from the extracellular to intracellular space $\left(X\to I\right)$ with rate constant ε
- *production decay*, an exponential decay $e^{-\text{κ}t}$ of production when transitioning from an expression pulse to chase at t=0, with rate constant κ representing first order decay of labeled amino acids; note this does not affect the constitutive secretion scenario or the pulse segment of the pulse-chase scenario

Model equations were constructed in piecewise fashion, with different rate terms depending on which processes were included in a given candidate model (Supplemental Table S1). Note that the model with all processes absent (M1) is equivalent to our original ODE model (Figure 1A), and the model with maturation delay only (M2) is equivalent to the base DDE model (Figure 2A), and equivalent to the model formulation used in our previous publication,^13^ but here with updated parameter values based on parameterization with additional experimental data.

### Simulation & Analysis Software.

We simulated differential equations in MATLAB version R2022a. We used R version 4.3.1 with RStudio release 2023.09.1 for all further data analysis and data visualization. See Supplemental References for the full list of packages and toolboxes used. All code is available at https://github.com/gillsignals/sFlt1-paper.

### Simulating constitutive secretion experiments.

We simulate experiments in which cells secrete sFLT1 constitutively into the extracellular medium for a defined period of time (Figure 1B, **top**). First, we run a pre-simulation, representing the time period of cell plating and culture before the experimental measurements, in which the cells come to a steady state. For this, we initialize intracellular and extracellular sFLT1 to $\left(I_{0},X_{0}\right)=\left(0,0\right)$, we set rate parameters to desired values, and we run the simulation to intracellular steady state $I_{SS}$ (defined as less than 0.5% change in I over 20h of simulation). These conditions are then used as the initial conditions for the simulation of the actual experiment, except that to represent media change at t=0, we reset the extracellular concentration X to 0 without changing the intracellular sFlt1 $\left(I=I_{SS}\right)$. We then run the simulation for either 24h or 72h, matching the specific experiments being simulated, reporting $\left(I,X\right)$ at 1-minute intervals.

### Simulating pulse-chase experiments.

We also simulate experiments in which labeled media is introduced briefly and then removed, in order to track secretion of newly-synthesized (and thus labeled) sFLT1 (Figure 1B, **bottom)**. For these pulse-chase experiments, we initialize the intracellular and extracellular sFLT1 to $\left(I_{0},X_{0}\right)=\left(0,0\right)$, set the parameters to desired values (including nonzero sFLT1 production rate constant α), and run the simulation for 20m from $t=\left(t_{pulse},0\right)$ to mimic synthesis of labeled sFLT1 by cells exposed to pulse media. We used the resulting system state as the initial condition for the remaining simulation, with X reset to 0 at t=0 without changing I to represent media change to unlabeled chase media. We then reduced ɑ to zero and continued the simulation for 10h, reporting $\left(I,X\right)$ at 1-minute intervals. For candidate models with exponential decay of production, the production rate during the chase was instead $\text{α}e^{-kt}$.

### Simulation algorithms.

For ODE models, we simulated equations using ode23s in MATLAB with an absolute tolerance of 10^−12^, relative tolerance of 10^−8^, initial step size of 10^−2^, and all species constrained to non-negative values. For the DDE and other candidate models, we simulated equations using dde23 in MATLAB with an absolute tolerance of 10^−4^, relative tolerance of 10^−3^, initial step size of 10^−2^, and any negative concentrations that emerge due to numerical approximations (which are always very small) are forced to 0.

### Experimental data for optimization.

We compiled three independently published datasets of the time course of sFLT1 secretion by cultured human umbilical vein endothelial cells (HUVECs) (Supplemental Table S2). To distinguish the three datasets, we will refer to them by the name of the first author in each case. Two of these studies quantified constitutive sFLT1 secretion after media change in the presence of minimal serum and no added perturbation: one using ELISA measurements of absolute sFLT1 concentration in conditioned media (“Hornig”^30^) and one using quantitative Western blots of sFLT1 in both conditioned media and cell lysate (“Kinghorn”^13^). The third study used a pulse-chase experiment to quantify newly-synthesized sFLT1 in both conditioned media and cell lysate with quantitative Western blots (“Jung”^22^). For the Jung and Hornig studies, summary data were extracted from published figures using the Figure Calibration function in ImageJ.^31^ Two data points were omitted: Hornig 3h (assumed below the limit of quantitation for ELISA) and Jung 10h (trend inconsistent with all other experimental data and all simulations). The Kinghorn data were available in original tabulated form; to give all datasets comparable weight in optimization, the mean values at each time point were used in datasets where multiple measurements per time point were available (mean and median values were comparable).

### Initial guesses for parameter values.

We ran optimization on each model starting from n=100 (ODE and candidate DDE models) or n=1000 (final DDE model) sets of initial guesses for parameter values. These initial values were randomly sampled from ranges (Supplemental Table S3) based on previous rate estimates of protein synthesis, transport, and degradation processes from experimental proteomic approaches^32–34^ and previous mechanistic models of VEGF receptor tyrosine kinases,^26,27^ including parameter values from our previous implementation of candidate model M2.^13^

### Optimization cost function.

For each dataset, we first converted the predicted X and I values (from the simulations) from units of #/cell to the units measured by the experimental assay. Then, for each observation in that dataset, we calculated the relative error between simulated and experimental results, (simulated value – experimental value)/(experimental value). Finally, we calculated the sum of squares of these errors, equally weighting the error of each data point. To minimize the cost function, we performed nonlinear least squares optimization using lsqnonlin in MATLAB. We bounded the search space for each parameter as shown in Supplemental Table S3.

### Scoring of candidate models.

Each candidate model was assigned a quality score using the corrected Akaike Information Criterion (AIC_C_):

(Eq. 5) $$n\;\log\;log\left(\frac{c}{n}\right)+2k+\frac{2k\left(k+1\right)}{n-k-1}$$

where n is the number of experimental data points, C is the cost of a given model calculated using the custom cost function, and k is the number of degrees of freedom (number of parameters + 1) for that model.^35,36^ The first two terms represent the uncorrected AIC, and the last term is a correction factor for small sample size because $\frac{n}{k}<40$.

### Sensitivity analysis.

Both local and global sensitivity analyses were performed on the DDE model starting from a baseline of the median optimized parameter set. Local univariate sensitivity of key output variables (such as sFlt1 concentrations) to key mechanistic parameters was calculated by running simulations with one parameter increased by 10%, and the relative change in the output variable compared to baseline. For global univariate sensitivity analysis, we ran simulations for a wide span of parameter values, each parameter in turn being scanned from 100x lower to 100x higher than the baseline value.

### Simulation of chemical inhibition or genetic downregulation.

To represent the response to the acute addition of a chemical inhibitor of one of the model processes, we simulate constitutive secretion as defined above, with the pre-simulation running the system to steady state using the median set of optimized parameters, but when media change is applied at t=0, we also adjust a single process parameter (α, β, γ, δ, or τ) using $p^{\prime }=\left(1-f\right)\;*\;p$ where p is the initial parameter value and f is the inhibitor’s effect, i.e. the fractional inhibition of that process. To represent longer-term system perturbation, e.g. due to genetic mutation of a system component or by expression downregulation of a trafficking protein via RNA interference days before an experimental time course, we again simulate constitutive secretion, this time running both the pre-simulation to steady state and the experimental simulation with a single process parameter adjusted as $p^{\prime }=\left(1-f\right)\;*\;p$, where again p is the initial parameter value and f is the magnitude of the downregulation, i.e. the fractional inhibition of that process. To compare the predicted effects of acute and chronic perturbations of trafficking mechanisms, we used experimental measurements of intracellular and extracellular sFLT1 following inhibitor treatment, expressed as a fraction of control (i.e. the absence of perturbation).^13^ Using our model, evaluated at a range of inhibition strengths, we estimated the fractional inhibition for each inhibitor of that inhibitor’s target process, i.e. the predicted parameter reduction required for the model to produce the same relative change in sFLT1 as experimentally observed.

## Results

### ODE model of sFLT1 secretion.

The ODE model (see Methods, Equations 1–2) includes rate terms for production, secretion, intracellular degradation, and extracellular degradation, described by four parameters (Figure 1A). A general analysis of the ODE model solution dynamics (Figure S1) is described in more detail in Supplemental Results. By setting the time derivatives to zero, we calculated theoretical steady state solutions:

(Eq. 6) $$I_{SS}=\frac{\text{α}}{\text{β}+\text{γ}}$$

(Eq. 7) $$X_{SS}=\frac{\text{β}}{\text{δ}}I_{SS}=\frac{\text{αβ}}{\text{δ}\left(\text{β}+\text{γ}\right)}$$

and characteristic times to half-maximal concentrations for intracellular and extracellular sFLT1 assuming first-order kinetics for secretion and degradation:

(Eq. 8) $$T_{50\_I}=\frac{ln\left(2\right)}{\text{β}+\text{γ}}$$

(Eq. 9) $$T_{50\_X}=\frac{ln\left(2\right)}{\text{δ}}$$

The derivations of Equations 6–9 are given in Supplemental Methods.

To calibrate the model parameter values to sFLT1 biology, we used optimization algorithms to fit simulation predictions of sFLT1 concentrations simultaneously to intracellular and extracellular sFLT1 concentration datasets from three independent studies of sFLT1 secretion in human umbilical vein endothelial cells (HUVECs): one pulse-chase time course in relative units (“Jung”^22^) and two constitutive secretion time courses, one in relative units (“Kinghorn”^13^) and one in absolute units (“Hornig”^30^) (Supplemental Table S2; Figure 1B). The pulse-chase secretion experiment provided particularly useful data for characterizing early secretion behavior, while the constitutive secretion experiments allowed for estimation of biologically relevant production parameter values. The optimization appeared at first to be well-constrained, but resulted in a set of solutions with identical cost (goodness of fit) that produced identical extracellular and normalized intracellular time courses (Figure 1C, n=100 superimposed lines) and yet differed in absolute intracellular sFLT1 amount over 2 orders of magnitude (Figures 1D–E). These optimized fits reproduced experimentally observed dynamics of constitutive sFLT1 secretion into culture media, save 2 outlier data points ($X_{72h}$ and $I_{24h}$) that were inconsistent with other experimental observations. However, this initial model could not recapitulate two clearly observed features of early sFLT1 secretion dynamics in the chase phase of the pulse-chase scenario: (1) maintenance or increase of intracellular sFLT1 level for several hours after production ends and (2) a multi-hour delay in extracellular sFLT1 accumulation.

### Delay differential equation (DDE) model of sFLT1 secretion.

To more accurately model the observed sFLT1 secretion dynamics, we converted our model to a system of two delay differential equations (DDEs) by introducing a fixed time delay between sFLT1 production and trafficking (Figure 2A; see Methods, Equations 3–4). This “maturation delay” represents the time required for newly synthesized intracellular proteins to traverse the secretory pathway, reach a mature form, and be sorted into vesicles for trafficking. This delay is particularly important in the pulse-chase scenario because only newly synthesized (and therefore labeled) proteins are experimentally measured, and so even though unlabeled sFLT1 made before the media change might be secreted sooner, the labeled sFLT1 will only be extracellularly secreted and observed after the full delay. The model with the delay would therefore only represent the labeled sFLT1, for comparison to the experimental data. Under this scenario, the rate terms for secretion and intracellular degradation are functions of intracellular sFLT1 concentration at time t−τ, for some time delay τ, rather than at time t as in the initial model. A general analysis of the DDE model solution dynamics (Figure S2) is described in more detail in Supplemental Results. Because at steady state $I\left(t-\text{τ}\right)=\left(t\right)$, this model has the same steady state properties as the ODE model (Equations 6, 7).

We optimized the DDE model parameters to the constitutive and pulse-chase secretion datasets similarly to the ODE model. The optimized DDE model generated pulse-chase dynamics that fit experimental data better than the ODE model, and fit the constitutive secretion data as effectively as the ODE model (Figure 2B). However, note that in the pulse-chase experiment, intracellular sFLT1 increased above its t=0 baseline at 2h before decreasing, and in this DDE model formulation there is no mechanism by which intracellular sFLT1 could increase after production stops at t=0. This inconsistency could suggest that our model overlooks one or more processes essential to describing the biology of sFLT1 secretion, or it could be a consequence of noise in the data, in which case adding parameters to the model risks overfitting the data.

### Testing alternate candidate models.

**Figure 3.**
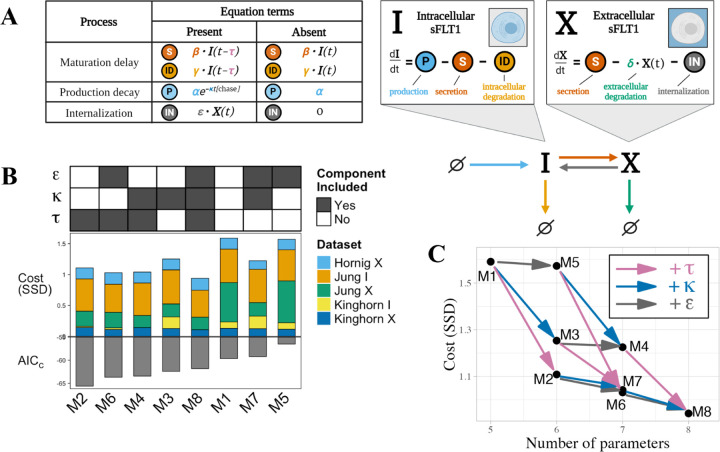
Comparing candidate models of sFLT1 secretion. **(A)** Structure of candidate model equations and equation term options for each process when present or absent in a given model. In the production decay term, the value of *[chase]* is 1 during the chase phase of pulse-chase simulations and 0 otherwise. **(B)** Top: Processes included (filled) or excluded (empty) in each model. ε: internalization, κ: production decay, τ: maturation delay. Middle: Cost (goodness of fit) of each model, defined as the sum of squared differences (SSD) between experimental data points and the lowest cost simulation of each model, with colors indicating the contribution of each experimental dataset. X: extracellular sFLT1, I: intracellular sFLT1. Bottom: Corrected Akaike Information Criterion (AIC_C_) scores for each model. **(C)** Impact of additional processes on the best-fit cost of the candidate models. Model nodes (points) are plotted as minimum model cost versus number of parameters. Edges (arrows) represent transitions between candidate model structures by adding the labeled process (τ (maturation delay): pink arrows, κ (production decay): blue arrows, ε (internalization): gray arrows. The vertical height of each arrow represents the decrease in cost from adding the labeled process to a given model.

We examined whether we could further improve our model by including different combinations of three processes: the maturation delay described above; first-order internalization of extracellular sFLT1; and exponential decay of production at the end of an expression pulse (see Methods). We defined rate terms for each reaction for cases when each process was present or absent; for example, the internalization process had a rate of ϵ⋅X when present but a rate of 0 when absent. Note that the absence of a process is equivalent to setting ε=0 (internalization), κ=0 (production decay), or τ=0 (maturation). Using these terms, we built an ensemble of eight models by generating each model’s equations in a piecewise fashion (Figure 3A, Supplemental Table S1). Note that the model with all additional processes absent (M1) is equivalent to our original ODE model (Figure 1A), and the model with maturation delay only (M2) is equivalent to the base DDE model (Figure 2A). We applied our optimization algorithm to each model with 100 different sets of initial parameters to compare the ‘best fit’ version of each model.

When considering only the lowest cost fit for each model, we found that all models with additional processes had a slightly improved fit (lower cost, defined as the sum of squared differences between simulated and experimental data points) relative to the original ODE model M1 (Figure 3B, middle). While models performed similarly in constitutive scenarios (Figure S3B, D), models that included maturation delay τ were better able to describe extracellular sFLT1 behavior in the pulse-chase scenario (Figure S3A), while inclusion of either maturation delay τ or production decay κ improved model fit to intracellular sFLT1 during pulse-chase (Figure S3C). Although every parameter addition improved goodness of fit, addition of maturation delay τ consistently caused the largest cost reduction (Figure 3C).

Improvements in fit required introducing additional parameters, so to reduce the risk of overfitting, we scored the models by balancing cost and complexity (number of parameters) using the corrected Akaike Information Criterion (AIC_C_, Equation 5).^35,36^ While adding maturation delay τ to any model reduced AIC_C_, adding production decay κ only reduced AIC_C_ if maturation delay τ is not already included, and adding internalization ε never reduced AIC_C_. Model M2, which includes maturation delay τ but not internalization ε or production decay κ and was analyzed in Figure 2, had the lowest AIC_C_ and thus had the best fit to the data given its number of parameters (Figure 3B: bottom). We interpret these results as showing that model M2 performs as well as the more complex models we tested based on the limited experimental data, so we therefore chose to move on to downstream analysis using this model (Figure 2A).

### Optimized parameter values.

To more thoroughly explore the properties of our selected DDE model, and to evaluate uncertainty in optimized parameter values for the model, we repeated the analysis with a larger set of 1000 optimization runs starting from randomly sampled initial values. While most resulting fits follow the experimental data well, numerous fits failed to converge to the experimental data (Figure S4A). To enrich for high-quality fits, we filtered for optimization runs where the cost of the resulting fit was within 10% of the minimum cost (n = 594) (Figure 2B, Figure S4B). Comparing the distribution of initial parameter values for all 1000 runs (Figure S4C, yellow; Supplemental Table S3) to the distribution of initial values for the 594 runs that passed the cost filter (Figure S4C, black), we can see that the filtered-out runs generally had extreme initial parameter values, especially large initial values of β.

**Figure 4.**
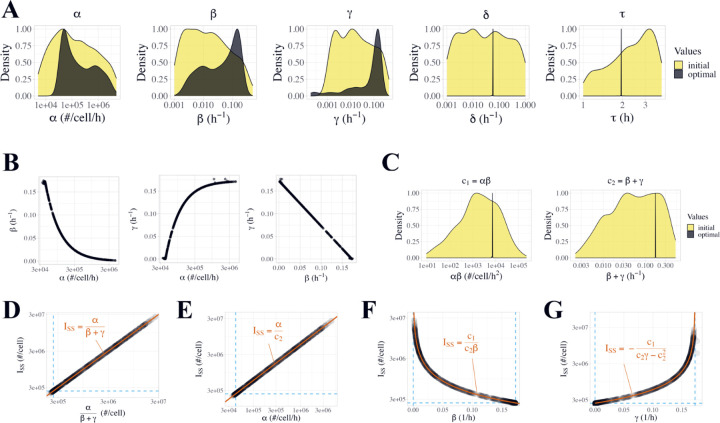
Properties of optimized parameter sets from the DDE model of sFLT1 secretion. **(A)** Distributions of initial (yellow) and optimized (black) model parameter values for the delay differential equation model after filtering for low-cost fits $\left(n=594\right)$. Y axes are normalized such that each distribution has a maximum density of 1. **(B)** Correlations between α, β, and γ values in optimized parameter sets. Each point represents the observed values of the listed parameters in a single run of the delay differential equation model after filtering for low-cost fits $\left(n=594\right)$. **(C)** Distributions of initial (yellow) and optimized (black) values of calculated compound parameters $c_{1}=\alpha \beta$ and $c_{2}=\beta +\text{γ}$ for the delay differential equation model after filtering for low-cost fits $\left(n=594\right)$. **(D-G)** Correlation between intracellular steady state sFLT1 $\left(I_{SS}\right)$ and **(D) $\alpha \text{/}\left(\beta +\text{γ}\right)$, (E) α, (F) β, (G) γ**. Dashed lines indicate theoretical bounds ($\min\left(I_{SS}\right)=c_{1}\text{/}c_{2}^{2}$, $\min\left(\alpha \right)=c_{1}\text{/}c_{2}$, $\min\left(\beta \right)=\min\left(\text{γ}\right)=0$, $\max\left(\beta \right)=\max\left(\text{γ}\right)=0$; see Supplemental Results). Bounds and highlighted trendlines are calculated using median values of $c_{1}$ and $c_{2}$ (Table 1).

After filtering, optimized values of δ and τ were very well-constrained (δ=0.057±0.00141/h, τ=1.958±0.020h), while the other parameters (α, β, γ) spanned 2–3 orders of magnitude (Figure 4A, Table 1, Figure S5A). Correlation plots show algebraic relationships between α, β, and γ, including an inverse nonlinear relationship between α and β, and an inverse linear relationship between β and γ (Figure 4B, Figure S5B). Because of these apparent relationships, we looked for joint constraints among combinations of parameters and found that both the product $c_{1}=\text{αβ}$ and the sum $c_{2}=\text{β}+\text{γ}$ are well-constrained to single values ($c_{1}=7252\pm 131\;\#{\text{/cell/h}}^{2}$, $c_{2}=0.173\pm 0.00064\;1\text{/h}$, mean ± SD) (Figure 4C, Table 2). This five-parameter model thus has four constraints (δ, τ, $c_{1}$, $c_{2}$) and only one degree of freedom: if any one of α, β, or γ is specified, then the values of the other two parameters are determined by the constants $c_{1}$ and $c_{2}$. When these constraints are present, Equations 6 and 7 can be extended as:

(Eq. 6a) $$I_{SS}=\frac{\text{α}}{\text{β}+\text{γ}}=\frac{\text{α}}{c_{2}}$$

(Eq. 7a) $$X_{SS}=\frac{\text{β}}{\text{δ}}I_{SS}=\frac{\text{αβ}}{\text{δ}\left(\text{β}+\text{γ}\right)}=\frac{c_{1}}{\text{δ}c_{2}}$$

### Steady state intracellular sFLT1.

During constitutive simulation, intracellular sFLT1 remains at a steady state $\left(I_{SS}\right)$ whose absolute amount varies across parameter sets within a range of 2.0 x 10^5^ - 2.6 x 10^7^ molecules per cell, with a median of 8.2 x 10^5^ #/cell (Figures S6). The simulated steady state values match the theoretical definition of $I_{SS}=\frac{\text{α}}{\text{β}+\text{γ}}$ across all fits (Figure 4D, Equation 6). Relationships between $I_{SS}$ and α, β, and γ also match the theory as follows. α is directly proportional to $I_{SS}$ (Figure 4E). β is inversely proportional to $I_{SS}$ and approaches a maximum value of $c_{2}$ as $I_{SS}$ approaches its minimum value (Figure 4F); as β increases, α decreases (because $c_{1}=\text{αβ}$) and thus $I_{SS}$ declines. γ is inversely related to β and approaches a maximum value of $c_{2}$ as β approaches 0; this causes α to increase, and so $I_{SS}$ approaches infinity (Figure 4G). The presence of the $c_{1}$ constraint explains why β and γ have opposite relationships to $I_{SS}$ despite both being in the denominator of $I_{SS}=\frac{\text{α}}{\text{β}+\text{γ}}$. Of note, each intracellular steady state is associated with a unique set of (α, β, γ) values, so high-confidence experimental measurements of intracellular sFLT1 would provide enough data to fully constrain our mechanistic model.

### Process fluxes.

**Figure 5.**
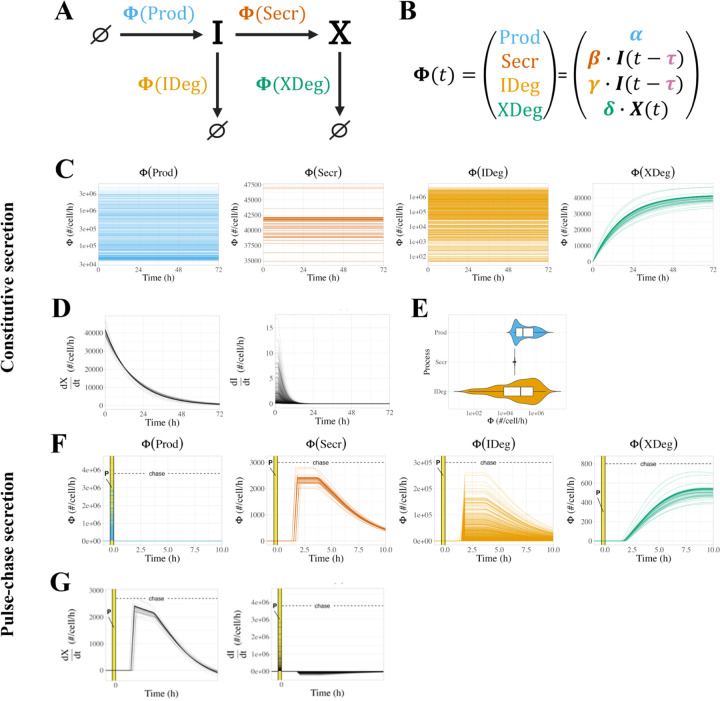
Analysis of simulated sFLT1 kinetic process fluxes. **(A)** Reaction network graph showing process fluxes $\left(\Phi \right)$. **(B)** Equations for process fluxes. **(C)** Process fluxes over time during simulation of constitutive sFLT1 secretion. **(D)** Rates of change of extracellular sFLT1 $\left(dX\text{/}dt=\Phi \left(\text{Secr}\right)-\Phi \left(\text{XDeg}\right)\right)$ and intracellular sFLT1 $\left(dX\text{/}dt=\Phi \left(\text{Prod}\right)-\Phi \left(\text{Secr}\right)-\Phi \left(\text{IDeg}\right)\right)$ during constitutive secretion. **(E)** Rates of change of extracellular sFLT1 $\left(dX\text{/}dt=\Phi \left(\text{Secr}\right)-\Phi \left(\text{XDeg}\right)\right)$ and intracellular sFLT1 $\left(dI\text{/}dt=\Phi \left(\text{Prod}\right)-\Phi \left(\text{Secr}\right)-\Phi \left(\text{XDeg}\right)\right)$ during constitutive secretion. **(F)** Constitutive flux distributions for time-constant processes. **(G)** Rates of change of extracellular and intracellular sFLT1 during pulse-chase secretion. In C, D, F, and G, each line represents a different optimized parameter set. Prod = production, Secr = secretion, IDeg = intracellular degradation, XDeg = extracellular degradation.

We explored the flux of each process, i.e. the rate of molecules traversing each edge of the reaction network graph (Figure 5A–B). During constitutive secretion, extracellular degradation flux increases hyperbolically over time, while the other three processes have constant fluxes over time (Figure 5C–D). The extracellular degradation flux converges towards a single time course directly proportional to the time course of extracellular sFLT1 (Figure 2B).

The secretion flux converges towards a single value of $\frac{c_{1}}{c_{2}}=\frac{\text{αβ}}{\text{β}+\text{γ}}$ across all model fits (Figure 5E, Figure S7A). In contrast, production and intracellular degradation flux values differ across fits and their values are paired such that any increased production flux is offset by intracellular degradation (Figure S7B), and both are algebraically related to the observed intracellular steady state concentration (Figure S7C–D, Supplemental Methods). The fit-specific differences in production and intracellular degradation flux also occur during simulation of pulse-chase secretion (Figure 5F–G), though all models had similar relative fluxes $\left(\frac{\Phi }{\Phi_{max}}\right)$ over time (Figure S8).

### Local univariate sensitivity analysis.

**Figure 6.**
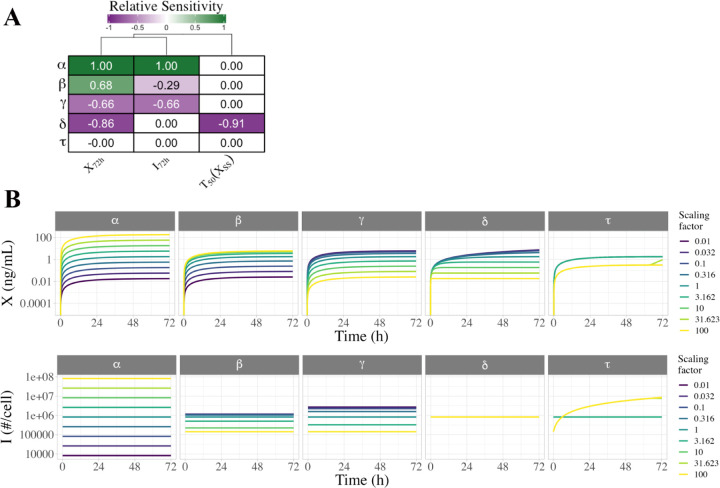
Local and global univariate sensitivity analysis of the sFLT1 secretion DDE model. **(A)** Relative sensitivity of sFLT1 secretion output variables to local changes in input parameters during simulation of constitutive secretion. The base case is the median parameter set, and all other cells represent the observed fractional increase in the output variable per fractional increase in the parameter. $X_{72h}$: extracellular sFLT1 after 72 hours; $I_{72h}$: intracellular sFLT1 after 72 hours; $T_{50\text{\_}X}$: time to half-steady-state for extracellular sFLT1. **(B)** Global univariate sensitivity analysis of extracellular and intracellular sFLT1 showing the effect of multiplying each input parameter by a scaling factor during constitutive secretion. The base case (scaling factor 1) is the median parameter set from the optimized parameter sets (Table 1).

We evaluated how uncertainty in each input parameter affects the uncertainty of selected output variables during constitutive secretion, including extracellular sFLT1 after 72h $\left(X_{72h}\right)$, intracellular sFLT1 ($I_{72h}$; note that for constitutive secretion, $I_{72h}=I_{SS}$), and time to half-steady-state for extracellular sFLT1 ($T_{50\text{\_}X}$ = time to $X_{SS}\text{/}2$), where $X_{SS}$ is calculated theoretically. Starting from the median parameter set (Table 1), we individually increased the value of each parameter by 10%, simulated constitutive secretion, and calculated the relative change in output variables (Figure 6A). Intracellular and extracellular sFLT1 increase linearly with production rate constant α (sensitivity = 1). Increasing the secretion rate constant β causes a moderate rise in extracellular sFLT1 but a mild decrease in intracellular sFLT1, while increasing the intracellular degradation rate constant γ causes a decline in both intracellular and extracellular levels. The extracellular degradation rate constant δ has a strong negative effect on extracellular sFLT1 but has no impact on intracellular protein. Notably, δ is the only parameter which affects the time to half-steady-state $\left(T_{50\text{\_}X}\right)$ for extracellular sFLT1, with higher δ associated with a faster time to steady state.

### Global univariate sensitivity analysis.

To explore how individual parameters affect sFLT1 trafficking over a larger range of values, we varied each parameter individually over several orders of magnitude and simulated constitutive secretion (Figure 6B, Figure S9). For all parameter sets tested, intracellular sFLT1 remains at its relevant steady state throughout a simulation, while extracellular sFLT1 increases hyperbolically towards a steady state. Consistent with the local sensitivity analysis, both extracellular and intracellular sFLT1 are most sensitive to changes in α, with changes in α causing directly proportional changes in both steady state intracellular sFLT1 and extracellular sFLT1 72 hours after media change, across the range of α values. β, γ, and δ have narrower ranges of effects than α (Figure 6B, Figure S9), because below or above certain thresholds changing the parameter value has no effect (Figure S9). While β and γ affect extracellular sFLT1 in opposite directions, they influence intracellular sFLT1 in the same direction but with different magnitudes, with the response to γ being larger and over a wider range than β. δ does not affect intracellular sFLT1, but decreasing δ increases extracellular sFLT1 while slowing its rate of convergence to steady state. The constitutive secretion scenario masks any impact of changing the secretion delay τ, with altered simulation results at high τ only because long delay interferes with our method of initializing the system to steady state before media change. While the constraints $\left(c_{1},c_{2}\right)$ are fixed for our sFLT1 system, we also explored the sensitivity of the system to changes in these values (Supplemental Results, Figure S10).

### Chemical perturbation of sFLT1 secretion.

**Figure 7.**
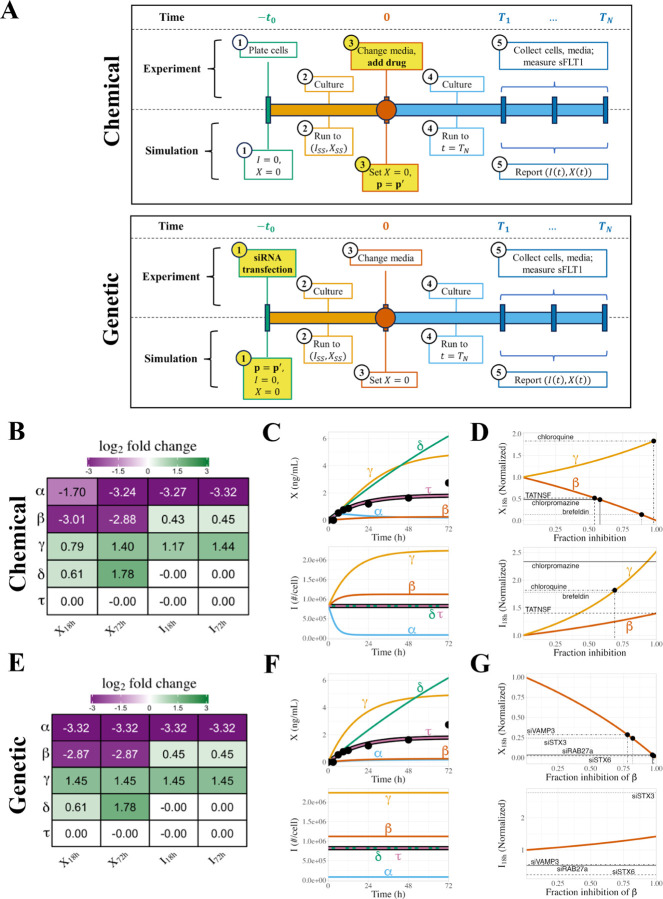
Simulated chemical inhibition and genetic downregulation of sFLT1 secretion. **(A)** Simulation timeline schematic for chemical inhibition (top) and genetic downregulation (bottom) of constitutive secretion. $p=p^{\prime }$ represents change of parameter values to apply inhibitor effect. **(B)** Effects of chemical inhibition of individual parameters by 90% on extracellular sFLT1 $\left(X\right)$ and intracellular sFLT1 $\left(I\right)$ at 18h and 72h, expressed as log2 fold changes relative to the control (uninhibited) case. **(C)** Time courses for absolute concentrations of extracellular $\left(\text{X}\right)$ and intracellular $\left(\text{I}\right)$ sFLT1 with 90% chemical inhibition of each parameter. The impact of inhibiting each process is how different each line is from the prediction of the median, unmodified parameter set (black line). Black points, experimental ELISA data for the uninhibited case (Hornig et al., 2000). **(D)** Annotated curves for chemical inhibition of β (red) and γ (orange) reproduced from (C). Horizontal lines indicate experimentally observed relative changes in extracellular $\left(X_{18h}\right)$ and intracellular $\left(I_{18h}\right)$ sFLT1 18 hours after drug addition. Vertical lines indicate the fraction inhibition of the target parameter required to reproduce experimental results. **(E)** Effects of genetic downregulation of individual parameters by 90%, as described in (B). **(F)** Time courses for absolute concentrations of extracellular $\left(\text{X}\right)$ and intracellular $\left(\text{I}\right)$ sFLT1 with 90% genetic downregulation of each parameter, as described in (C). **(G)** Annotated curves for genetic downregulation of β (red) and γ (orange), as described in (D).

We explored how our model of sFLT1 secretion responds to either chemical inhibition or genetic downregulation of various processes during constitutive secretion. Chemical inhibition represents the application of a druglike inhibitor at a specific experimental time point, while genetic downregulation reflects a system where genes encoding cellular trafficking machinery are mutated or their expression is blocked by RNAi before the experimental time course begins (Figure 7A). Chemical inhibition is acute, genetic downregulation more chronic; thus, to simulate chemical inhibition, we first ran the simulation to steady state with baseline parameter values, and then we applied media change $\left(X\to 0\right)$ and inhibitor treatment (parameter value adjustment) at t=0, while to simulate genetic downregulation, we applied the parameter changes continually from the beginning of the simulation, before running the simulation to steady state.

Starting from the median parameter set (Table 1), we chemically inhibited each parameter individually by 90% and investigated how inhibition affects intracellular and extracellular sFLT1 over time (Figure 7B–C). Inhibition of the production rate constant α causes a gradual but large decrease in intracellular sFLT1, which approaches a new lower steady state within 16h, while extracellular sFLT1 initially increases before declining to a new steady state within 48h. Inhibition of the secretion rate constant β causes a gradual small increase in intracellular sFLT1, which approaches a new higher steady state within 16h, while extracellular sFLT1 hyperbolically approaches steady state with lower concentration but similar dynamics as the uninhibited case. Inhibiting the intracellular degradation rate constant (γ) leads to a hyperbolic increase in both intracellular and extracellular sFLT1, both of which take over 48h to approach steady state. Inhibiting extracellular degradation (δ) does not alter intracellular sFLT1 but causes extracellular sFLT1 to increase continuously throughout the entire 72h simulation. Adjustment of the secretion delay time (τ) does not influence sFLT1 during constitutive secretion.

We then evaluated the impact of strength of inhibition on extracellular and intracellular sFLT1 at two time points: 18 hours, a time point at which inhibitors of sFLT1 secretion have been experimentally tested,^13^ and 72 hours, the standard length of our constitutive simulation. Consistent with previous findings, inhibiting production (α) decreases both intracellular and extracellular sFLT1, inhibiting secretion (β) slightly increases intracellular sFLT1 but strongly decreases extracellular sFLT1, inhibiting intracellular degradation (γ) increases both intracellular and extracellular sFLT1, and inhibiting extracellular degradation (δ) does not affect intracellular sFLT1 but increases extracellular sFLT1 (Figure S11A). All these effects increase in size as fraction inhibition increases, although the effect of inhibiting α increases linearly with fraction inhibition and the effects of inhibiting other parameters increase superlinearly. While the effects of inhibiting α and β are roughly equivalent at 18h and 72h, the impact of inhibiting γ and δ are larger at the later time point, consistent with only the α and β-perturbed simulations approaching their new steady state by 18h.

We compared our model predictions to previous experiments^13^ evaluating how chemical inhibitors of the secretory pathway affect intracellular and extracellular sFLT1 after 18h of treatment (Figure 7D, Table 3). Based on observed experimental changes in extracellular sFLT1, our model suggests the lysosomal degradation inhibitor chloroquine inhibits the sFLT1 intracellular degradation rate constant γ by 98%, while the observed change in intracellular sFLT1 suggests a lower but still substantial 69% inhibition of γ. Similarly, based on extracellular sFLT1 experimental measurements, our model suggests the Golgi inhibitor brefeldin inhibits the sFLT1 secretion rate constant β by 89%, while the vesicular transport inhibitor chlorpromazine and the exocytosis inhibitor TATNSF700 only inhibit β by 58% and 54% respectively. However, the experimental intracellular sFLT1 concentrations observed for all 3 drugs exceeded the predicted concentration expected by our model even if secretion is fully blocked. This suggests our model may be missing an important component describing intracellular retention of sFLT1, as inhibiting secretion leads to much larger accumulation of intracellular sFLT1 than our model can explain. This could also suggest that the effect of secretion-inhibiting drugs is more complex than simply altering the secretion rate constant, possibly also reducing intracellular degradation, e.g. by relocating sFLT1 to subcellular compartments from which degradation does not occur.

### Genetic perturbation of sFLT1 secretion.

Genetic inhibition of individual parameters (Table 4) by 90% produced similar results as chemical inhibition at later time points (Figure 7E), but dynamics of early inhibition differed in many cases due to the presence of inhibition during the pre-simulation stage (Figure 7F); for example, intracellular sFLT1 remains at steady state after media change at t=0, but this steady state level is reduced by inhibiting the production rate constant α or increased by inhibiting the secretion rate constant β or intracellular degradation rate constant γ. The steady state intracellular levels induced by 90% genetic inhibition are the same as the intracellular levels approached over time during 90% chemical inhibition, but because the genetic inhibitor is applied earlier, these steady state levels have already been reached by t=0. Extracellular sFLT1 time courses for genetic and chemical inhibition are identical for inhibition of β, γ, and the extracellular degradation rate constant δ. Genetic inhibition of α causes extracellular sFLT1 to hyperbolically approach its decreased steady state level, unlike in chemical inhibition where extracellular sFLT1 initially overshoots its final level before a delayed decrease to the new steady state.

Genetic parameter inhibition has the same directional effects on extracellular and intracellular sFLT1 at 18h and 72h as in chemical inhibition (Figure S11B). The impact of inhibiting extracellular degradation (δ) is larger at the later time point, with the discrepancy increasing in size as fraction inhibition increases. For all other parameters, genetic inhibition led to the same changes in intracellular and extracellular sFLT1 at both 18h and 72h.

We compared our model predictions to previous experiments^13^ exploring how small interfering RNA (siRNA) inhibition of the secretory pathway affects intracellular and extracellular sFLT1 (Figure 7G; Table 4), assuming that the inhibition reached its maximum effectiveness by the media change at t=0 and remained fully effective throughout the remaining experimental time. Based on observed experimental changes in extracellular sFLT1, our model suggests that inhibition of the tested Golgi and vesicular trafficking machinery (STX6, RAB27a, STX3, VAMP3) decreases the effective secretion rate constant β by 78–98%, consistent with these factors playing a role in sFLT1 secretion. However, while our model predicts a minor increase in intracellular sFLT1 with inhibition of the secretion rate constant, genetic inhibition of STX3 leads to a much larger intracellular sFLT1 accumulation than expected by our model even if secretion is fully blocked, and genetic inhibition of the other tested factors caused a paradoxical decrease in intracellular sFLT1. These observations again suggest our model is an incomplete descriptor of intracellular sFLT1 biology, despite model agreement with other observed experimental data; for example, the targets of inhibition can affect the trafficking of many proteins, which may themselves have a role in sFLT1 trafficking.

### Evaluating uncertainty: simulating inhibition using different initial parameter sets.

Since there is some remaining uncertainty in parameter values, we explored how this uncertainty impacts the above insights into chemical and genetic inhibition. We generated five parameter sets consistent with the observed constraints, by setting the baseline secretion rate constant β to one of five different values ($0.2*c_{2}$, $0.4*c_{2}$, $0.6*c_{2}$, $0.8*c_{2}$, $c_{2}$), then calculating $\text{α}=c_{1}\text{/β}$ and $\text{γ}=c_{2}-\text{β}$ for each case. To reiterate, these parameter sets all satisfy the underlying temporal data; so our question was: would comparing simulations for different parameter sets to the inhibitor data help us to distinguish which parameter set or sets are best? Thus, for each parameter set (Figure S12), we tested the effect of chemical or genetic inhibition by a range of inhibition fractions at both 18h and 72h (Figure S13). In chemical inhibition, the effects of inhibiting sFLT1 production (α) or extracellular degradation (δ) are independent of the initial parameter set, but the choice of parameter set affects the size (though not the direction) of the system’s response to inhibiting either sFLT1 secretion (β) or intracellular degradation (γ) (Figure S13A). As β increases, intracellular sFLT1 becomes more sensitive to inhibiting β and less sensitive to inhibiting γ. Extracellular sFLT1 response to γ inhibition also depends on the initial parameter set, while the initial parameter set has a smaller impact on extracellular sFLT1 response to β inhibition and effects differ only at intermediate β inhibition fractions. These differences between parameter sets are more pronounced at 72h than at 18h. For genetic inhibition, simulated results resemble later time points for chemical inhibition simulations for the intracellular parameters α, β, and γ (Figure S13B). Extracellular sFLT1 is more sensitive to increasing fraction inhibition of β or γ at 72h than at 18h, but this effect is independent of the initial parameter set, and for all other parameters the results of genetic inhibition match at these two time points.

**Figure 8.**
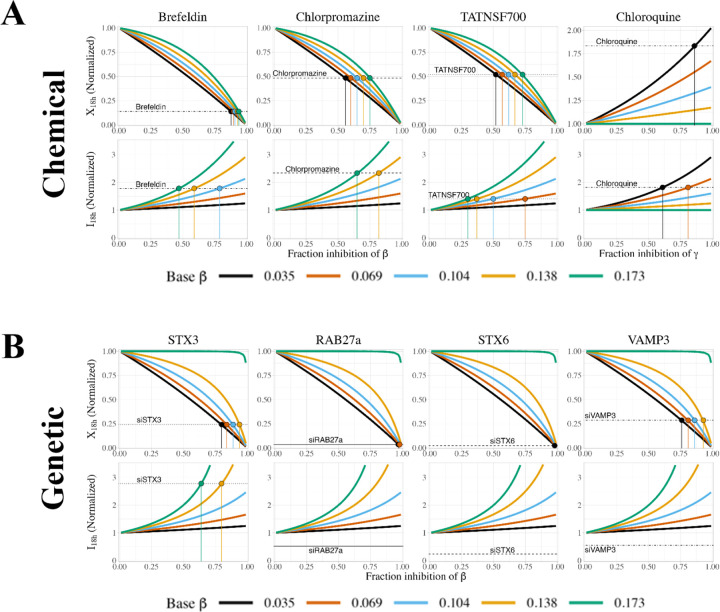
Simulating chemical and genetic inhibition of sFLT1 secretion from different initial parameter sets. **(A)** Annotated curves for chemical inhibition of β (secretion, first 3 columns) and γ (intracellular degradation, rightmost column) at 18 hours. Horizontal lines indicate experimentally observed relative changes in extracellular $\left(X_{18h}\right)$ and intracellular $\left(I_{18h}\right)$ sFLT1 18 hours after addition of brefeldin, chlorpromazine, TATNSF700, or chloroquine. Colored vertical lines mark the fraction inhibition required to match the observed change in sFLT1 when starting from the parameter set with the matching β value. **(B)** Annotated curves for genetic inhibition of β (secretion rate constant) at 18 hours post media change. Horizontal lines indicate experimentally observed relative changes in extracellular $\left(X_{18h}\right)$ and intracellular $\left(I_{18h}\right)$ sFLT1 after 18 hours after media change in cells transfected with siRNA targeting the listed secretion-linked protein 48 hours previously.^13^ Colored vertical lines mark the fraction inhibition required to match the observed change in sFLT1 when starting from the parameter set with the matching β value.

We then compared our inhibition simulations to experimental results^13^ (Figure 8). Based on extracellular sFLT1 data from chemical inhibitors, all parameter sets generated plausible estimates (<100%) for inhibition fraction of secretion (β), with higher fraction inhibition estimated for higher initial β values (i.e. lower intracellular sFLT1 levels), and with more uncertainty in the effect size for the moderate inhibitors chlorpromazine and TATNSF700 (55–75%) than for the strong inhibitor brefeldin (88–95%) (Figure 8A). In contrast, for intracellular sFLT1 following chemical inhibitors, the estimated β inhibition fraction increases as β decreases (intracellular sFLT1 increases), and a feasible fraction inhibition of β (<100%) can only be estimated for parameter sets with initial β values above a certain threshold, which differs for each inhibitor (approximately, β>0.5 for TATNSF700, β>0.7 for brefeldin, and β>1.1 for chlorpromazine). When initial values of β are low, even full inhibition of β does not generate a large enough fold change in intracellular sFLT1 to match experimental results. This suggests an additional constraint on the value of the secretion rate constant β. The intracellular degradation (γ) inhibitor chloroquine shows the opposite pattern: for both intracellular and extracellular sFLT1, the estimated γ inhibition fraction increases as β increases (intracellular sFLT1 decreases), and a feasible fraction inhibition of γ (≤100%) can only be estimated at lower initial values of β (and thus high initial values of γ and intracellular sFLT1), approximately β>0.1. Collectively, these suggest that an intermediate value of β, similar to the median value of 0.072 (Table 1), can provide a basis for most inhibitor observations; our earlier results showed that a constrained value of β also constrains α and ${\text{I}}_{\text{SS}}$.

Comparing simulated and experimental genetic inhibitors of β (Figure 8B), we find that all parameter sets except those with the highest initial β values (lowest intracellular sFLT1) led to reasonable (<100%) estimates of inhibition fraction, again consistent with an intermediate β value. However, experimental downregulation of the secretion factors VAMP3, RAB27a, and STX6 decreased intracellular sFLT1, whereas in our simulations, inhibiting secretion increased intracellular sFLT1 for all inhibition fractions. Only experimental STX3 inhibition changed intracellular sFLT1 by a magnitude consistent with our simulations, and only for parameter sets with high initial values of β>0.11 (low intracellular sFLT1). Our interpretation is that these inhibitors may have mechanistic effects different to those included in our model – such as effects on the trafficking on other proteins that themselves impact sFLT1, as mentioned above – and bear further investigation.

## Discussion

Here, we have developed mechanistic computational models of sFLT1 secretion by endothelial cells. sFLT1 is a soluble form of the VEGFR1 protein, whose membrane-associated form is one of three receptor tyrosine kinases that bind VEGF family cytokines, a key family of ligands that induces blood vessel growth in health and disease. Because sFLT1 can bind the ligand but has no tyrosine kinase domain, it does not initiate signaling and therefore acts as a sink for ligand, downregulating its signaling potential. Therefore, sFLT1 is an important negative regulator of blood vessel growth, and understanding its production, secretion, and levels outside the cell are important to understanding VEGF regulation.

There have been a limited number of experimental studies that quantified sFLT1 secretion over time by endothelial cells,^13,22,30,37^ and here we have used the data on sFLT1 levels inside and outside the cell from several of those studies to parameterize the models. We also used data from a recent study^13^ that tested multiple chemical and genetic inhibitors of proteins known to be involved in protein trafficking and secretion, to determine which were relevant for sFLT1 secretion, and in so doing quantified the level of disruption to sFLT1 extracellular levels over time (and therefore sFLT1 secretion) due to these inhibitors. Since the inhibitors block or reduce the effectiveness of the mechanisms included in our models, we explored model perturbations equivalent to these inhibitors in the simulations also.

A key first step in our study was comparing different mechanistic models for sFLT1 secretion. These models differed in the incorporation or exclusion of mechanisms such as: a maturation time delay between production and availability for secretion; a gradual decay in production rate when production ceases; and the internalization of previously-secreted extracellular sFLT1. The addition of some of these time-based processes also requires shifting from a typical ODE-based model to a DDE (delay differential equation)-based model. The resulting eight candidate models were tested, i.e. the parameters for each were optimized independently against the three experimental datasets described earlier and the predictions of the best-fit parameter sets for each model then compared across the eight models. The criterion used for overall model choice was the Akaike Information Criterion (AIC), which balances goodness of fit (i.e. how closely the simulation predictions match the experimental data) with the complexity of the model (i.e. how many parameters are needed to define it, with the understanding that more parameters would require more data for determination). As a result, model M2 - a DDE-based model that included maturation delay but not internalization or production decay - was identified as the best model and was used for the remainder of our study. This does not mean that production decay and internalization do not happen in the actual cells, nor indeed that they don’t improve goodness of fit of the model when included in the simulations; rather, it means that their effect is relatively small and the improvement is not worth the additional model complexity.

Based on this chosen model, and comparing the results of hundreds of optimization runs attempting to fit to the experimental data, of the five mechanistic parameters (α, β, γ, δ, τ), two of them (δ, extracellular degradation; τ, maturation delay) are very well constrained. These parameters are likely well-constrained because they are integral to the shape of the concentration curves over time, something that is relatively invariant to the total amount of sFLT1 in the system; this is relevant because much of the experimental data is not in absolute concentration units, and thus the comparisons between simulation and experiment are done after both are normalized to be on the same scale. In the absence of an absolute intracellular sFLT1 measurement, the other parameters (α, β, γ) are not fully constrained. Thus, we have a family of solutions - however, we observed that these α, β, and γ values are not all independent, and two further constraints are apparent when comparing the optimized parameter sets: $c_{1}=\text{αβ}$ (i.e. production rate times secretion rate constant is a fixed value), and $c_{2}=\text{β}+\text{γ}$ (i.e. the sum of secretion and intracellular degradation rate constants is a fixed value). As a result, there is only one degree of freedom remaining, and setting any one of these three parameter values (or knowing the value of intracellular sFLT1 levels) determines the other parameters. Thus, even in the absence of a single completely unique parameter set, the resulting model of sFLT1 is still well defined because (a) the overall dynamics are fairly consistent due to the constraint of δ and τ, and (b) the additional constraints on α, β, and γ mean that there is a unique solution for any given intracellular sFLT1 level, i.e. if it were possible to measure an absolute value for intracellular sFLT1 levels, then the system would be fully determined. Experiments that measure the dynamic responses to selective perturbations (e.g. of intracellular degradation) may help pin down the specific rate constants; we also explore the uncertainty in model predictions due to different parameter sets that are consistent with the experiments and span the range of possible parameter set values.

We use the parameterized model to simulate chemical inhibitors (e.g. small molecules that interfere with key trafficking processes) and genetic inhibitors (e.g. small interfering RNAs (siRNA) that downregulate the expression of key proteins involved in trafficking), in particular a subset of the inhibitors that were experimentally tested in a recent study.^13^ The inhibitors are simulated in the model by the modification of the rate parameters for the process that the inhibitors are thought to interfere with; for example, if the inhibitor blocks intracellular degradation, the value of γ is decreased. Note that there are known to be many sequential steps to secretion in cells, and the inhibitors tested included those targeting different molecules at different steps, but in our model we aggregate all secretion steps to one process. We distinguish between chemical and genetic inhibitors in our model based on when we apply the change in parameter values: to simulate chemical inhibition, we allow the system to reach steady state with baseline parameters before adjusting any parameter values, whereas we simulate genetic inhibition by adjusting the target parameter from the start of the simulation. This timing difference – longer-term genetic inhibition vs acute chemical inhibition – may give time for genetic inhibition to result in second-order effects where proteins and processes not included in the model are affected and can then themselves impact sFLT1 trafficking.

Overall, we see mixed results when comparing our simulations to the experimental observations. Our model is consistent with the experimental data for all tested chemical inhibitors of secretion and intracellular degradation for at least some of the possible parameter sets tested. For example, the secretion inhibitor brefeldin decreases extracellular sFLT1 and increases intracellular sFLT1, and our simulations predict the drug decreases the secretion rate constant by over 50% and likely closer to 90%, the precise value depending on which parameter set is used. However, the experimental data for several genetic inhibitors behave unexpectedly and change in the opposite direction compared to our model. For example, we simulate inhibiting the vesicular trafficking factor VAMP3 as decreasing the secretion rate constant while leaving other parameters unchanged, which is predicted to decrease extracellular sFLT1 while increasing intracellular sFLT1; but experimental downregulation of VAMP3 instead decreases intracellular sFLT1. Furthermore, this mismatch occurs regardless of which initial conditions we choose from the family of optimized parameter sets.

It is not immediately clear how to interpret or resolve this discrepancy between our model and experiments for these genetic inhibitors. It is difficult to identify a single missing process that, if included in the model, would explain lower intracellular sFLT1 when secretion is blocked. Rather, as noted above it is more likely that there is some complex regulation at play for those specific inhibitors. Importantly, the chemical and genetic inhibitors explored are not specific to sFLT1, but regulate trafficking for many other proteins, some of which may themselves modulate sFLT1 synthesis and secretion. Furthermore, nonspecific trafficking inhibitors like brefeldin can cause massive accumulation of immature protein in the ER and Golgi, and the resulting cell stress can lead to the unfolded protein response (UPR), a complex homeostatic response with multifactorial effects on the secretory pathway. Among its many effects, the UPR increases protein turnover, and while global protein synthesis slows during the UPR, there is increased transcription and translation of secretory pathway components affecting protein maturation and trafficking.^38^ As such, any inhibitor that triggers the UPR could have second-order effects altering the rate constants for sFLT1 production, trafficking, and degradation. Cases like this, where the experimental perturbations affect multiple model parameters and invoke dynamic feedback regulation, would be difficult to parse in this model. Focusing on acute inhibition or measuring changes as soon as feasible after inhibition may better serve to avoid second-order effects. What the model does do, however, is identify precisely those inhibitors whose observed behavior/outcome does not match what would be expected to happen, and therefore provides a useful shortlist of inhibitors whose more detailed mechanisms and effects would be interesting to follow up on.

Returning again to the remaining uncertainty in the model parameter values, we explored whether the inhibitor experiments could help to pin down the model parameters. Each drug has a different set of parameter ranges for which modeling inhibition is well-behaved, however these ranges are not all consistent; for example, some drugs seem to support a high-β-value parameter set, others a low-β-value parameter set. An intermediate β-value is the most broadly consistent with experimental data, but further model development, perhaps including more detailed inhibitor mechanisms, may better constrain our parameter estimates.

Our results provide mechanistic insights about sFLT1 secretion in vitro and suggest intriguing questions about intracellular behavior of sFLT1. It remains to be seen whether these insights would also apply in vivo; if so, it suggests that physiological or pathological changes in sFLT1 concentration could have multiple causes. For example, elevated serum sFLT1 (analogous to extracellular sFLT1 in our model) could result from an increase in sFLT1 production, or increased secretion, or decreased intracellular or extracellular degradation, or any combination of these changes. Different disorders are likely driven by distinct changes in sFLT1 kinetics, with implications for treatment approaches. In vivo conditions are further complicated by multi-cellular expression and secretion of sFLT1; for example, in preeclampsia, non-endothelial cells in the placenta overexpress sFLT1 mRNA^21^. These models could be extended to understand the effects of such multicellular environments.

Interestingly, our model suggests that less than 20% of mature intracellular sFLT1 is removed from the intracellular compartment each hour under basal conditions, consistent with a previous estimate^22^ and corresponding to a long intracellular residence time for sFLT1. This extended residence time raises the question of whether sFLT1 has functional VEGF-binding and VEGFR-binding roles within the secretory pathway. Interactions can happen because the full-length receptors accumulate in the same intracellular locations. The full-length mFLT1/VEGFR1 isoform localizes primarily to the Golgi and traffics to the plasma membrane in a calcium-dependent manner,^8^ while VEGFR2 is largely retained in the trans-Golgi and traffics to the cell surface in response to VEGF stimulation.^39^ RTKs are also known to initiate signals from intracellular organelles^7–10^ as well as from their more canonically considered cell surface locations, and this may also occur within the Golgi and be inhibited by Golgi-resident sFLT1.

Our approach identifies a minimal mechanistic model consistent with experimental data on sFLT1 secretion, but includes many assumptions that simplify the underlying biology, both for general features of the secretory pathway and for specific characteristics of sFLT1. The key assumptions involve homogeneity and the choice of model compartments, species, and reactions, each described in the following paragraphs. This mass action kinetics modeling approach uses several assumptions of homogeneity, including that all sFLT1 molecules in a compartment are identical and equally likely to react, all cells are identical with the same rate constants, and that system geometry is constant over space and time. However, biological systems are highly heterogeneous. Tissues consist of numerous cell types of different lineages that vary significantly in their gene expression and phenotypic profile, typically arranged into higher-order three-dimensional structures that are not spatially uniform. Individual cell types occupy various states and can change states over time. Even in monolayer culture of single cell lines, cells are not identical, as the stochasticity of biochemical reactions leads to natural gradients and fluctuations.

Furthermore, here we assume a constant number of cells over time when simulating or interpreting experimental results, while the cell culture environment allows cell growth and proliferation. Since HUVECs proliferate slowly in culture,^40^ this is likely not a major confounder at early time points, but cell number may have increased enough by our 72 hour time point to impact data interpretation. A hierarchical model that includes both sFLT1 trafficking and cell population dynamics may add insight and reveal how changing cell number affects interpretation of the experimental data.

An agent-based modeling (ABM) approach could incorporate cells of different types with distinct phenotypes and reaction propensities, simulate stochasticity, represent cell proliferation, and track spatial heterogeneity with partial differential equations. However, ABMs tend to be computationally expensive, we may not have sufficient data for parameterization, and their complexity may not be necessary to understand key features of sFLT1 secretion in cell culture. An intermediate approach that allows modeling of heterogeneous cell communities without the full computational demand or spatial detail would be to use ODEs to model several separate intracellular compartments (corresponding to cell subtypes) linked to a common extracellular compartment. However, since sFLT1 expression varies not only across cell types but across adjacent endothelial cells,^20^ it may be more appropriate to use a spatially-aware modeling approach like an ABM to explore heterogeneous cell populations. While ABMs have been extensively applied to the biology of the VEGF family,^41–45^ some including extracellular sFLT1,^46,47^ to our knowledge they have not been used to explore intracellular sFLT1 behavior and secretion.

In our model, all locations for sFLT1 are aggregated into only two compartments for sFLT1: intracellular and extracellular. In reality, both consist of several subcompartments where molecules can have different levels and be exposed to different conditions and therefore have different reaction propensities. For example, the intracellular space represents the entire endomembrane system of organelles (such as ER, Golgi, vesicles, and endosomes). While traversing these organelles, intracellular sFLT1 undergoes a series of maturation and trafficking steps that make a newly synthesized molecule more likely to be secreted over time, meaning reaction propensities are not constant. Although our final model addresses this maturation problem by including a maturation delay (τ) in between synthesis and secretion or degradation, protein maturation is likely more variable and stochastic than can be represented with a fixed time delay. sFLT1 could also localize to noncanonical compartments; both VEGFR1 and VEGFR2 have been shown to localize to the nucleus under certain conditions in endothelial and microvascular cells.^48^ Outside the cell, since sFLT1 binds HSPGs in the extracellular matrix (ECM) or on the cell surface, and also dimerizes with membrane-integral VEGFR isoforms, extracellular sFLT1 can also be subdivided into cell-surface-associated, ECM-associated, and freely soluble forms. Explicitly representing these subcompartments or maturation states would add new molecular species and reactions, increasing model complexity and increasing the range of possible behaviors in our model. However, they would also have significantly more parameters, and estimating those parameter values would require quantitative data about sFLT1 organelle distribution or post-translational modification. As a separate but related issue, our optimization to experimental data assumes that all extracellular sFLT1 is in conditioned media and that protein detected in cell lysates is intracellular. However, extracellular sFLT1 bound to ECM may not be aspirated with conditioned media, and extracellular sFLT1 associated with the cell surface (either dimerized to membrane VEGFR or nonspecifically bound to HSPGs) could be isolated with the lysate.

Our current model assumes that only sFLT1 affects the system, that we can omit other molecules and reactions from our model, and that the processes we do model are well-described by zeroth- and first-order kinetics with fixed rate constants. This framework does not address all the complexity of cell biology; for example, factors affecting global gene expression, such as ribosomal subunit expression and nutrient availability, could dynamically alter the sFLT1 production rate. Similarly, other secretory pathway cargo interacts with shared trafficking machinery, so high production of other secretory proteins could reduce the trafficking factors available to sFLT1 and reduce the effective secretion rate constant β.

sFLT1 binds other intracellular and extracellular components, which not only mediate its effects on VEGF signaling but also can affect the dynamics we depict on our model, as bound sFLT1 may not be available for trafficking reactions. sFLT1 acts as a ligand trap for extracellular VEGF and PlGF, and also potentially as a dimerization partner for membrane-integral VEGF receptor isoforms that creates dominant negative receptor complexes.^12^ These networks of biochemical reactions may have emergent properties inconsistent with our assumption of invariant rate constants. For example, VEGF-A upregulates sFLT1 expression, which then sequesters VEGF and reduces VEGF signaling.^37,49^ This negative feedback loop could generate pulses of sFLT1 expression or oscillations,^50^ either of which would change the sFLT1 production rate over time. Similar feedback effects could also affect proteins involved in secretion or degradation. Alternatively, some evidence suggests sFLT1 can be generated by proteolytic cleavage of membrane-integral VEGFR1,^51^ a potential second production route for sFLT1, and a model incorporating that reaction would also need to track membrane isoforms and potentially be expanded to the level of gene expression and FLT1/VEGFR1 RNA alternative splicing. sFLT1 also contains heparin-binding domains that nonspecifically bind glycoproteins on the ECM and cell surface^52–54^ and accumulates in the ECM.^55^ sFLT1-ECM binding is important for establishing and maintaining extracellular VEGF gradients that are directional cues in angiogenesis.

Importantly, protein glycosylation occurs in intracellular secretory pathway organelles, especially the Golgi,^56^ and interaction with intracellular glycoproteins is a potential mechanism for sFLT1 retention in the Golgi. Adding such extensive additional detail to the model would require substantial amounts of additional specific experimental data, and would likely not impact on the core observed secretion dynamics as reproduced by the current model.

These caveats and considerations noted above can provide further directions for development of the model of sFLT1 secretion developed here. Even without these additions, the model is already well-behaved, recreates most experimental observations of sFLT1 secretion, and identifies two classes of inhibitors or perturbations: those whose behavior is well represented by the model, and those whose behavior seems contrary to the model and worthy of further investigation.

## Supplementary Material

Supplement 1

## Figures and Tables

**Table 1.** Statistics for the distribution of optimized values of core model parameters (n=594 parameter sets).

parameter	α	β	γ	δ	τ
**unit**	#/cell/h	1/h	1/h	1/h	h
**mean**	5. 177×10^5^	7. 229×10^−2^	1. 005×10^−1^	5. 725×10^−2^	1. 958
**SD**	7. 692×10^5^	6. 411×10^−2^	6. 419×10^−2^	1. 444×10^−3^	2. 046×10^−2^
**CV**	1. 487	8. 868×10^−1^	6. 389×10^−1^	2. 523×10^−2^	1. 045×10^−2^
**median**	1. 419×10^5^	5. 123×10^−2^	1. 215×10^−1^	5. 743×10^−2^	1. 958
**MAD**	1. 470×10^5^	6. 639×10^−2^	6. 649×10^−2^	2. 361×10^−5^	1. 570×10^−2^
**IQR**	6. 310×10^5^	1. 337×10^−1^	1. 337×10^−1^	3. 149×10^−5^	2. 097×10^−5^
**median/IQR**	4. 447	2. 609	1. 1	5. 483×10^−4^	1. 071×10^−5^
**min**	3. 524×10^4^	1. 592×10^−3^	1. 842×10^−4^	4. 577×10^−2^	1. 695
**max**	4. 563×10^6^	1. 745×10^−1^	1. 767×10^−1^	7. 296×10^−2^	2. 197

SD = standard deviation, CV = coefficient of variation, MAD = median absolute deviation, IQR = interquartile range.

**Table 2.** Statistics for the distribution of compound parameters $c_{1}=\text{αβ}$ and $c_{2}=\text{β}+\text{γ}$ resulting from the optimized parameter values (Table 1).

parameter	$c_{1}=\text{αβ}$	$c_{2}=\text{β}+\text{γ}$
**unit**	#/cell/h^2^	1/h
**mean**	7. 252×10^3^	1. 728×10^−1^
**SD**	1. 316×10^2^	6. 418×10^−4^
**CV**	1. 814 × 10^−2^	3. 715×10^−2^
**median**	7. 269×10^3^	1. 727×10^−1^
**MAD**	1. 207	2. 302×10^−7^
**IQR**	1. 606	3. 145×10^−7^
**IQR/median**	2. 210 ×10^−4^	1. 821×10^−6^
**min**	5. 999×10^3^	1. 685×10^−1^
**max**	8. 460×10^3^	1. 835×10^−1^

SD = standard deviation, CV = coefficient of variation, MAD = median absolute deviation, IQR = interquartile range.

**Table 3.** Predicted inhibition strengths of chemical inhibitors based on comparing experimental data^13^ to model simulations.

Treatment	Inhibited process	Parameter affected	Percent inhibition in model to match	
			$X_{18h}$	$I_{18h}$
brefeldin	Golgi assembly and maturation (Misumi et al., 1986; Fujiwara et al., 1988; Lippincott-Schwartz et al., 1989)	β	89	> 100*
chlorpromazine	clathrin-mediated vesicle trafficking (Wang et al., 1993)	β	58	> 100*
TATNSF700	exocytosis; vesicle fusion (Matsushita et al., 2005)	β	54	> 100*
chloroquine	lysosomal degradation (Mauthe et al., 2018; Lie and Schofield, 1973)	γ	98	69

* Simulations match the direction of the experimental perturbation but cannot achieve the observed strength of impact.

**Table 4.** Predicted inhibition strengths of genetic inhibitors based on comparing experimental data^13^ to model simulations.

Treatment	Target role	Detailed role	Parameter affected	Percent inhibition in model to match	
				$X_{18h}$	$I_{18h}$
siRAB27a	vesicle trafficking to plasma membrane	small GTPase coordinating vesicle transport along actin (Wu et al., 2001; Alzahofi et al., 2020)	β	97	< 0^†^
siSTX3	vesicle trafficking to plasma membrane	t-SNARE at plasma membrane (Sharma et al., 2006)	β	82	> 100*
siSTX6	vesicle trafficking from Golgi	t-SNARE in trans-Golgi network (Bock et al., 1997; Wendler and Tooze, 2001)	β	98	< 0^†^
siVAMP3	vesicle trafficking to plasma membrane	v-SNARE in endosome-derived vesicles (Hu et al, 2007)	β	78	< 0^†^

* Simulations match the direction of the experimental perturbation but cannot achieve the observed strength of impact.

† Simulations cannot match experimental data as experimental perturbation has opposite outcome to simulated perturbation.
